# Concurrent chemoradiotherapy with cisplatin + S-1 versus cisplatin + other third-generation agents for locally advanced non-small-cell lung cancer: a meta-analysis of individual participant data

**DOI:** 10.1186/s12890-022-01828-z

**Published:** 2022-01-09

**Authors:** Yuri Taniguchi, Hiroaki Okamoto, Tsuneo Shimokawa, Tomonari Sasaki, Takashi Seto, Seiji Niho, Yuichiro Ohe, Yusuke Saigusa

**Affiliations:** 1 Department of Respiratory Medicine, Yokohama Municipal Citizen’s Hospital, 1-1 Mitsuzawa-nishimachi, Kanagawa-ku, Yokohama, Kanagawa 221-0855 Japan; 2 Department of Clinical Radiology, Graduate School of Medicine, Kyushu University, Fukuoka, Japan; 3 Department of Thoracic Oncology, National Hospital Organization Kyushu Cancer Center, Fukuoka, Japan; 4 Department of Pulmonary Medicine and Clinical Immunology, Dokkyo Medical University, Tochigi, Japan; 5 Department of Thoracic Oncology, National Cancer Center, Tokyo, Japan; 6 Department of Biostatistics, Yokohama City University School of Medicine, Yokohama, Japan

**Keywords:** S-1, Vinorelbine, Pemetrexed, Docetaxel, Locally advanced non-small-cell lung cancer, IPD meta-analysis

## Abstract

**Background:**

For decades, concurrent chemo-radiotherapy with cisplatin-based regimen has been a standard therapy for locally advanced stage III non-small-cell lung cancer (NSCLC). We conducted individual-participant-data (IPD) meta-analyses to compare S-1/cisplatin versus other third-generation anti-cancer medications plus cisplatin regimens with the goal of determining whether or not S-1/cisplatin was the ideal choice for treatment accompanied by radiotherapy (RT).

**Methods:**

A thorough search was performed using multiple electronic databases. We integrated the IPD of each trial and analyzed the resulting meta-database. The primary endpoint was the overall survival (OS), and the secondary endpoints included the progression-free survival (PFS), objective response rate (ORR), toxicities, and treatment delivery. Subgroup analyses were conducted based on baseline characteristics. Statistical analyses were stratified by trials.

**Results:**

Three randomized control trials (WJOG5008L study, SPECTRA study, and TORG1018 study) were found. Of the 316 patients enrolled in those studies, 159 received S-1/cisplatin (SP), and 157 were assigned to other combination chemotherapy. The median OS for the SP arm was 48.2 months, and that of the non-SP arm was 42.4 months. The combined hazard ratio (HR) for the OS was 0.895 (95% confidence interval [CI] 0.638–1.256), and no heterogeneity was noted among the trials (test for heterogeneity, p = 0.87; *I*^2^ = 0). The median PFS for the SP and non-SP arms was 12.8 and 14.0 months, respectively. The corresponding HR for the PFS was 1.022 (95% CI 0.776–1.347), and there was evidence of moderate heterogeneity among the trials (test for heterogeneity, p = 0.16; *I*^2^ = 0.46). The ORRs were 69.7% (95% CI 62.1–76.7%) and 70.9% (95% CI 63.7–78.1%) in the SP and non-SP arms, respectively. The toxicity profile showed that SP caused significantly fewer instances of grade 3–4 leukopenia and neutropenia than non-SP regimens.

**Conclusion:**

No marked differences were detected in the OS, PFS, or ORR between the SP and non-SP arms. SP had significantly less myelosuppression and better treatment compliance as a chemotherapy regimen for concurrent chemoradiation in locally advanced NSCLC than non-SP regimens.

**Supplementary Information:**

The online version contains supplementary material available at 10.1186/s12890-022-01828-z.

## Introduction

Patients diagnosed with stage III locally advanced non-small-cell lung cancer (NSCLC) account for approximately 30% of all lung cancer patients. Concurrent chemoradiotherapy is the only way to potentially achieve a cure in cases where radical surgery cannot be performed. In 1968, thoracic radiotherapy (RT) alone was shown to be associated with a better overall survival (OS) than best supportive care. However, the median survival time was only 9 months, and the 5-year survival rate was only 5% [1]. A meta-analysis published in 1995 discussed concurrent chemoradiotherapy, which was associated with a significantly improved survival when combined with cisplatin regimens [2].

Since third-generation chemotherapy is preferred to second-generation chemotherapy when combined with cisplatin, as reported in several trials in the 1990s, many studies have also targeted stage III locally advanced NSCLC treated with chemoradiotherapy, including the OLCSG007 and PROCLAIM studies [3, 4]. The OLCSG007 study compared mitomycin + vindesine + cisplatin (MVP), which was considered to be a standard regimen at the time, with cisplatin + docetaxel, and the two-year survival rate of CDDP/DTX was significantly better than that of MVP. Cisplatin + etoposide and cisplatin + pemetrexed were subsequently compared in cases of non-squamous NSCLC in the PROCLAIM study. Although that study failed to prove the superiority of cisplatin + pemetrexed in the OS, this regimen was shown to be associated with markedly less hematological toxicity than cisplatin + etoposide.

In the present clinical setting, cisplatin + vinorelbine, cisplatin + docetaxel, cisplatin + S-1, weekly carboplatin + PTX (paclitaxel) [5], and daily carboplatin for NSCLC and cisplatin + pemetrexed for non-Sq NSCLC are the options available. Recently, the PACIFIC study reported the promising treatment of stage III NSCLC with the anti-PD1 inhibitor durvalumab as consolidation therapy following concurrent chemoradiotherapy [6]. We strongly believe that determining the most appropriate chemotherapy regimen to accompany thoracic RT is of the utmost importance for keeping up with the fast-changing environment of stage III NSCLC treatment.

S-1, a third-generation oral fluoropyrimidine agent, consists of a mixture of tegafur, gimeracil, and oteracil potassium at a molar ratio of 1:0.4:1. Gimeracil maintains a high 5-FU level by inhibiting the degradation enzyme, whereas oteracil reduces gastrointestinal toxicity by inhibiting the 5-FU activity in the gut. The efficacy and safety of this regimen were highlighted in two phase-3 trials related to advanced-stage NSCLC [7, 8]. We found several clinical trials comparing S-1-containing regimens combined with RT to other regimens for locally advanced stage NSCLC performed in the last decade. Performing an integrated analysis of these findings may lead to the identification of new therapeutic prospects.

The main objective of this systemic review and meta-analysis based on individual participant data (IPD) was to compare CDDP + S-1 with CDDP + other third-generation anticancer agents combined with thoracic RT for stage III locally advanced NSCLC, with a statistical power much higher than that of either trial separately.

## Material and methods

### Identification of eligible trials

A literature search was performed in December 2019 to identify all published and unpublished randomized trials comparing S-1 to other third-generation anti-cancer agents combined with cisplatin for stage III NSCLC. The search was conducted using electronic databases, such as PubMed, Medline, and the Cochrane library, as well as major international conferences, such as the American Society of Clinical Oncology (ASCO), European Society of Medical Oncology (ESMO), and World Congress on Lung Cancer (WCLC). The clinical trials.gov website was also checked thoroughly. The following keywords were used: ‘stage III locally advanced’, ‘non-small cell lung cancer’, ‘S-1’, and ‘cisplatin’.

### The data collection and database quality

IPD were requested from each data center for all patients enrolled in all identified trials thanks to the efforts of the principal investigators. Data from each individual study were checked and verified for coherence with the original publications. The quality of the database was reassured for all eligible studies.

### IPD

The following IPD were requested for all randomly assigned patients: age, gender, performance status at the time of the enrollment, smoking history, histopathology, staging, allocated treatment, radiotherapy, site of primary tumor, survival, response to treatment, distant recurrence, treatment compliance, and adverse events graded using the National Cancer Institute-Common Terminology Criteria for Adverse Events (NCI-CTCAE). Data were checked for missing values, validity, and consistency, and the missing values were not supplemented. We also ensured that there were no imbalances or unusual patterns in treatment assignment or baseline characteristics in each trial. Any questions were discussed and solved with each data center/trial investigator.

### Statistical analyses

The analyses were performed on all randomized patients meeting the inclusion criteria and receiving the actual treatment according to the modified intention-to-treat principle. The modified intention-to-treat patient population included everyone who received the study treatment at least once and did not violate the eligibility criteria and had been analyzed for relapse and the survival. The safety population was defined as all patients receiving at least one course of protocol drugs. A fixed-effect model was used to obtain a summary of each trial’s treatment effect on the OS/progression-free survival (PFS) and assess the heterogeneity among them. The I^2^ and Cochrane’s Q tests were also performed to investigate the variation percentage and heterogeneity.

The primary endpoint was the OS, which was defined as the time from　randomization until death. The secondary endpoints were the PFS, response rate, and toxicity rate. The PFS was defined as the time from the randomization to progression or death, whichever occurred first. Survival curves (PFS and OS) were drawn by the Kaplan–Meier technique. The median follow-up time was assessed according to the Kaplan–Meier method. The relative effect of each treatment arm in different subgroups was investigated using the same stratified analyses. Once analyses were performed for each subgroup sorted by the age, performance status, gender, clinical staging, histology, and smoking status, these results were then combined to give overall hazard ratios (HRs) for the SP and non-SP regimens. Furthermore, a multivariate analysis was performed using the Cox proportional hazards regression model to assess whether or not each variable of the patients’ characteristics and treatment choice influenced the OS. All categorical variables were analyzed by the chi-square test or Fisher’s exact test. The differences in the mean values of the relative dose intensity in the two arms were evaluated by Student’s *t*-test. All p-values were two-sided, and a p-value < 0.05 was considered to indicate a statistically significant difference. Analyses were carried out using the JMP software program; version 15.0, SAS; version 9.4, and R; version 4.0.3 (SAS Institute Inc., Cary, NC, USA).

## Results

### Characteristics of the trials

Ten trials were identified, among which seven were not eligible (three were designed as single-arm trials [9–11], one was retrospectively examined [12], two compared SP to cisplatin alone [13, 14], and one used uracil/tegafur instead of S-1 [15]). We identified three phase-2 randomized clinical trials, all conducted in Japan: the WJOG5008L [16], SPECTRA [17], and TORG1018 [18] studies. The main characteristics of the three trials are described in Table 1. CDDP/S-1 was compared to CDDP/VNR, CDDP/PEM, and CDDP/DTX in the WJOG5008L study, SPECTRA study, and TORG1018 study, respectively. Only the SPECTRA study restricted the inclusion criteria to non-Sq NSCLC, whereas the other two included all NSCLCs.

**Table 1:** Characteristics of the three randomized controlled trials included in the meta-analysis

	WJOG5008L	SPECTRA	TORG1018
Regimen	TRT + S-1 + CDDP vs TRT + VNR + CDDP	TRT + D-1 + CDDP vs TRT + PEM + CDDP	TRT + S-1 + CDDP vs TRT + DTX + CDDP
N	108 (54 each)	102 (52 vs. 50)	106 (53 each)
RT (Gy)	60	60	60
primary endpoint	2-year OS rate	2-year PFS rate	2-year OS rate
randomization period	Sep/2009 to Sep/2012	Jan/2013 to Oct/2016	May/2011 to Aug/2014
follow-up period (months)	44.6	37.3	41.7
Eligibility criteria			
Age (years)	20–74	20–74	20–74
PS	0 or 1	0 or 1	0 or 1
Stage	Unresectable stage III	Unresectable stage III	Unresectable stage III
HR for OS (95% CI)	0.85 (0.48–1.49)	0.95 (0.53–1.74)	0.87 (0.49–1.55)
Median OS (95% CI)	40.9 (61.7–85.0) vs 39.0 (54.3–79.1)	48.3 (32.3-NR) vs 59.1 (24.1–65.6)	55.2 (32.7-NR) vs 50.8 (30.1-NR)
Median PFS (95% CI)	14.8 (10.7–18.4) vs 12.3 (10.2–14.3)	12.7 (9.46–17.55) vs 13.8 (7.85–16.39)	11.8 (9.5–17.1) vs 19.9 (12.3–29.9)

RT, radiotherapy; TRT, thoracic radiotherapy; CDDP, cisplatin; VNR, vinorelbine; PEM, pemetrexed; DTX, docetaxel; HR, hazard ratio; OS, overall survival; PFS, progression-free survival; NR, not reached; CI, confidence interval

In the SP arm, S-1 at 40 mg/m^2^ was administered twice daily on days 1–14 following cisplatin infusion at 60 mg/m^2^ on day 1. The actual dosage of S-1 according to the body surface area (BSA) was as follows: BSA < 1.25 m^2^, 80 mg daily; BSA 1.25 m^2^ to < 1.50 m^2^, 100 mg daily; and BSA ≥ 1.5 m^2^, 120 mg daily. The WJOG5008L and TORG1018 studies had the same administration protocol; SP administration was repeated every four weeks during the RT period and three weeks during the consolidation period. In the SPECTRA study’s protocol, SP was administered every four weeks the entire time. The CDDP/VNR arm received vinorelbine 20 mg/m^2^ on days 1 and 8 and cisplatin 80 mg/m^2^ on day 1. In the CDDP/DTX arm, docetaxel 50 mg/m^2^ and cisplatin at 80 mg/m^2^ were administered on day 1. Both CDDP/VNR and CDDP/DTX were administered on the same schedule; the first two courses of CCRT were repeated every four weeks, and the remaining two courses were given every three weeks as consolidation therapy. The CDDP/PEM regimen consisted of intravenous cisplatin at 75 mg/m^2^ and pemetrexed 500 mg/m^2^ on day 1 simply repeated every 3 weeks. Concurrent TRT was initiated on day 1 of the first cycle of chemotherapy. The planned total dose was 60 Gy, fractioned 30 times, for 2 Gy per day for 6 weeks. Three-dimensional conformal radiotherapy (3DCRT) was applied in all three trials, with no intensity-modulated radiotherapy (IMRT) cases included.

### Patients’ characteristics and treatment outcomes

#### Patients’ characteristics

A total of 316 patients were included in the identified trials, with 159 patients undergoing S-1-based regimens and 157 assigned to other third-generation anti-cancer drug regimens (non-SP). The baseline characteristics of the 316 eligible patients are described in Table 2. The median age of the patients in the SP and non-SP arms was 63 (range: 39 to 74) years old and 64 (range: 32 to 74) years old, respectively. The proportion of male patients was higher than that of female patients in both arms (SP: 74.8%, non-SP: 75.1%). Regarding histology, adenocarcinoma was found in more than half of each group (SP: 68.6%, non-SP: 66.2%). Further characteristics, such as the detailed histology, clinical staging, smoking history, and Eastern Cooperative Oncology Group Performance Status (ECOG PS), showed no significant differences between the two arms.

**Table 2:** Baseline patients’ characteristics of each group

	SP (n = 159)	Non-SP (n = 157)	p value
Gender, n (%)			
Male	119 (74.8%)	118 (75.1%)	1.000
Female	40 (25.2%)	39 (24.9%)	
Age, median (range)	63 (39–74)	64 (32–74)	0.690
≥ 70	27	35	
< 70	132	122	0.259
Stage, n (%)			
IIIA	84 (52.8%)	78 (49.7%)	0.653
IIIB	75 (47.2%)	79 (50.3%)	
Histological type			
Adenocarcinoma	109 (68.6%)	105 (66.9%)	0.810 (adeno versus non-adeno)
Squamous cell carcinoma	31 (19.5%)	28 (17.8%)	
Adenosquamous cell	1 (0.6%)	0 (0%)	
NOS	15 (9.4%)	21 (13.4%)	
Others	3 (1.9%)	3 (1.9%)	
Smoking history			
Never	29 (18.2%)	26 (16.6%)	0.767
Current/former	130 (81.8%)	131 (83.4%)	
PS			
0	103 (64.8%)	94 (59.9%)	0.417
1	56 (35.2%)	63 (40.1%)	

SP, S-1 + cisplatin; NOS, not otherwise specified; PS, performance status

#### Treatment delivery

A total of 121 (76.1%) and 124 (78.9%) patients tolerated the targeted 4 cycles of chemotherapy in the SP and non-SP arms, and 152 (95.5%) and 153 (97.4%) patients completed 60 Gy RT, respectively (Table 3). In addition, there were 149 (93.7%) and 150 (95.5%) patients in the SP and non-SP arms who completed RT within 56 days from the start. Of the patients who received more than 2 courses of chemotherapy, a dose reduction was needed in 26 (17.9%) and 42 (27.4%) (p = 0.0493) in the SP and non-SP arms, respectively, and a delay in the treatment course was seen in 114 (78.6%) and 97 (63.4%) (p = 0.0049), respectively (Table 3). The relative dose intensity (RDI) was calculated separately for each drug during the concurrent chemoradiotherapy period and consolidation period. The average RDIs of CDDP for the SP and non-SP arms during the induction chemoradiotherapy period were 91.3% (95% confidence interval [CI] 89.4–93.2%) and 95.1% (95% CI 93.2–97.0%) (p = 0.007), and those during the consolidation period were 69.5% (95% CI 63.7–75.3%) and 74.9% (95% CI 69.6–80.2%) (p = 0.173), respectively. The average RDIs for S-1 and other third-generation anti-cancer drugs were 90.5% (95% CI 88.3–92.7%) and 93.7% (95% CI 92.2–95.3%) (p = 0.019) during the induction phase and 67.8% (95% CI 62.1–73.5%) and 73.8% (95% CI 68.6–79.0%) (p = 0.129) during the consolidation phase, respectively (Additional file 1: Table S1). The reasons for the treatment course delay showed no significant difference between the arms (Additional file 1: Table S2).

**Table 3:** Treatment delivery

	SP (n = 159)	Non-SP (n = 157)	χ^2^ test P-value
Chemotherapy			
1	14	4	
2	18	17	
3	6	12	
4	121 (76.1%)	124 (78.9%)	
RT			
< 40 Gy	4	0	
40–59 Gy	3	4	
60 Gy	152 (95.5%)	153 (97.4%)	
Median (range)	60 (16–60)	60 (40–60)	
Completed RT within 56 days	149 (93.7%)	150 (95.5%)	
More than 2 courses of chemotherapy	145	153	
Dose reduction	26 (17.9%)	42 (27.4%)	0.049
Delayed course	114(78.6%)	97(63.4%)	0.005
Relapse	108	110	
Subsequent therapy following relapse	100 (92.5%)	89 (80.9%)	0.010

SP, S-1 + cisplatin; RT, radiotherapy

#### The OS and PFS

**Fig. 1:**
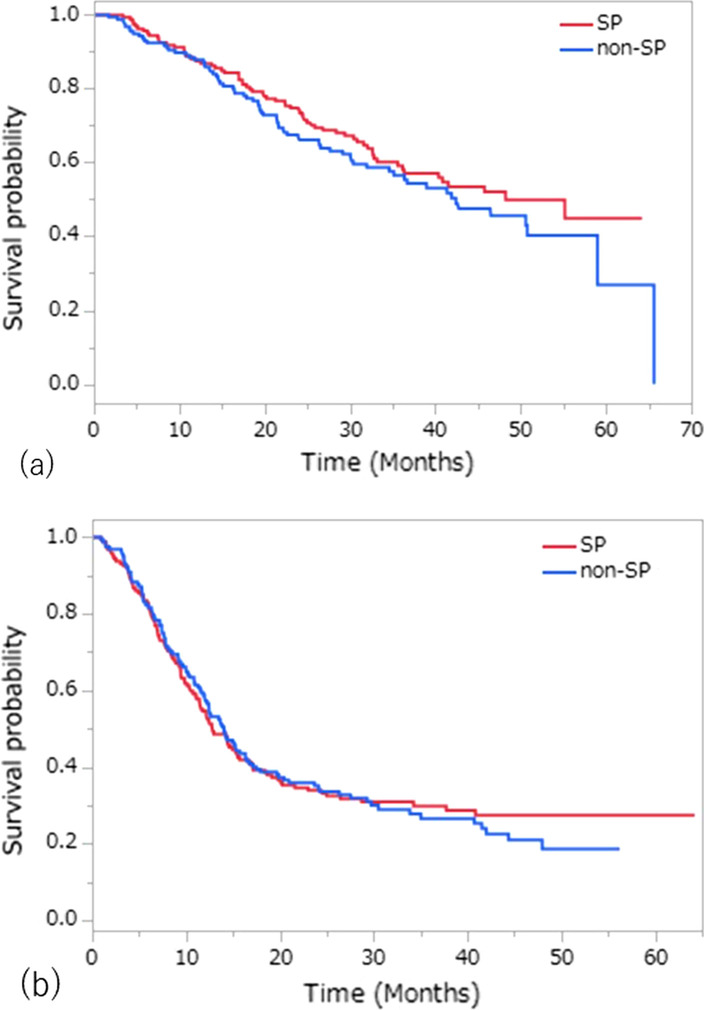
**a** The overall survival (OS) and **b** progression-free survival (PFS) curves by treatment arm

**Fig. 2:**
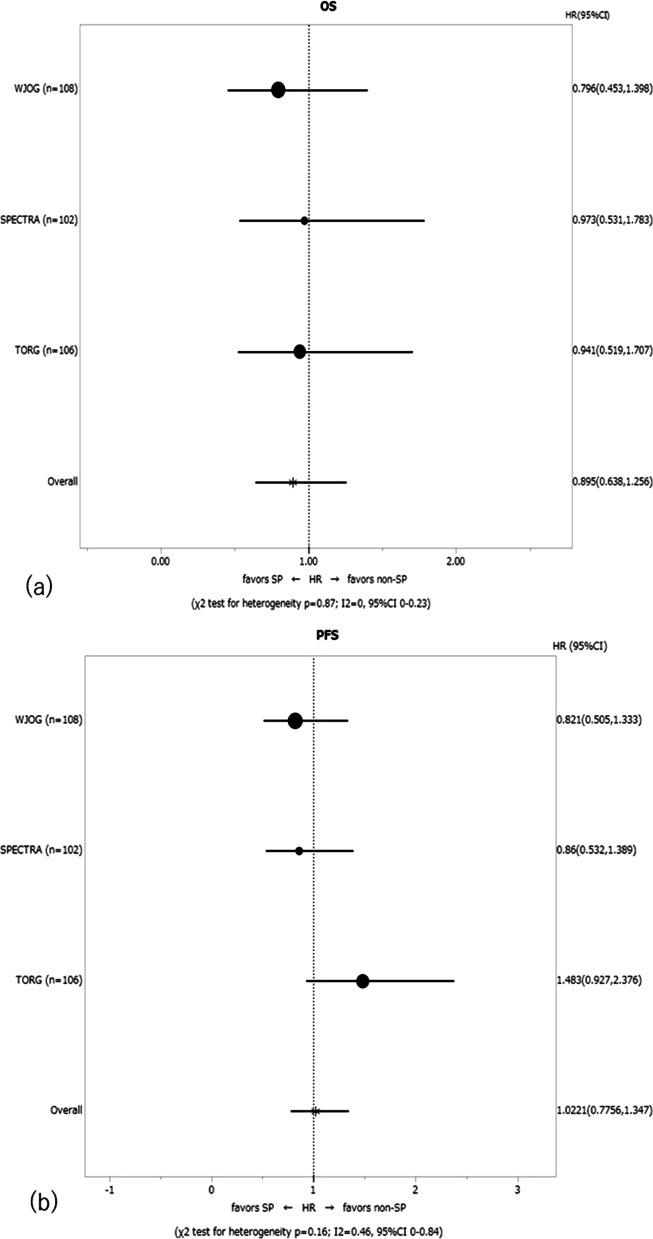
Forest plot of **a** the overall survival (OS) and **b** progression-free survival (PFS) by trial

**Fig. 3:**
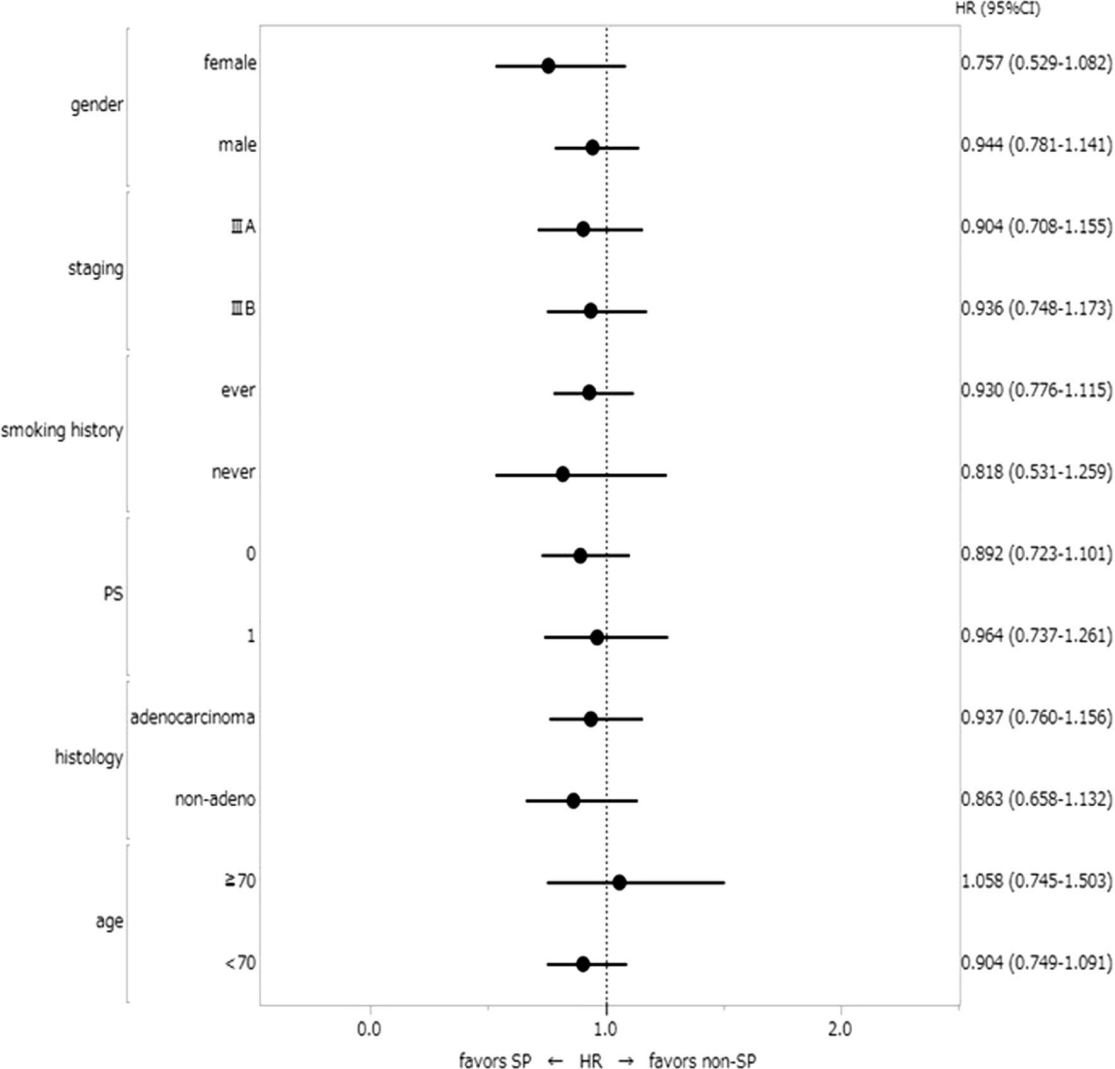
Forest plot of the subgroup analysis for the overall survival

The OS and PFS curves for patients according to the assigned treatment are shown in Fig. 1. Overall, there were 143 deaths (68 in the SP arm, 75 in the non-SP arm), and the median OS was 48.2 and 42.4 months in the SP and non-SP arms, respectively. The 2-year OS rates were 74.7% and 67.5%, and the 5-year OS rates were 44.9% and 26.9% in the SP and non-SP arms, respectively (Additional file 1: Table S3). Although the SP arm exceeded the non-SP arm with regard to the OS curve, there was no statistically significant difference. The combined HR of the OS was 0.895 (95% CI 0.638–1.256), with no heterogeneity noted among the trials (χ^2^ test for heterogeneity p = 0.87; *I*^2^ = 0, 95% CI 0–0.23) (Fig. 2a). Subgroup analyses showed no factors among patients’ baseline characteristics that affected the difference in the OS between the SP and non-SP arms (Fig. 3). In the multivariate analysis with all patients’ baseline characteristics and the heterogeneity of each trial taken into account, the HR of SP treatment compared to non-SP was 0.881, showing no statistically significant difference (p = 0.454). An age ≥ 70 years old was significantly associated with a worse OS than an age < 70 years old (HR, 1.638; p = 0.016) (Additional file 1: Table S4).

During the follow-up period, a total of 218 cases of progression (108 in the SP arm, 110 in the non-SP arm) were observed. The median PFS and 2-year PFS rate in the SP arm were 12.8 months and 33.9%, respectively, while those values for the non-SP arm were 14.1 months and 35.2%, respectively. No significant difference in the PFS was noted between the groups. The corresponding HR for the PFS was 1.022 (95% CI 0.776–1.347), and there was evidence of moderate heterogeneity among the trials (χ^2^ test for heterogeneity p = 0.16; *I*^2^ = 0.46, 95% CI 0–0.84) (Fig. 2b).

#### Response

Since data on two patients from each group were not obtained in the WJOG5008L study, the response analysis was based upon 157 patients in the SP arm and 155 patients in the non-SP arm. The objective response rates (ORRs) in the SP and non-SP arms were 69.7% (95% CI 62.1%-76.7%) and 70.9% (95% CI 63.7%-78.1%), respectively, and the disease control rates (DCRs) were 92.3% (95% CI 88.1%-96.5%) and 94.1% (95% CI 90.4%-97.9%), respectively (Table 4). There was no significant difference between the groups in the ORR or DCR.

**Table 4:** Response

	SP (n = 157)	Non-SP (n = 155)	p-value
Best response			
CR	2	4	
PR	107	106	
SD	36	36	
PD	8	7	
NE	4	2	
ORR			
CR + PR	109 (69.4%)	110 (70.9%)	0.431
(95% CI)	(62.1–76.7)	(63.7–78.1)	
DCR			
CR + PR + SD	145 (92.3%)	146 (94.1%)	0.337
(95% CI)	(88.1–96.5)	(90.4–97.9)	

SP, S-1 + cisplatin; CR, complete response; PR, partial response; SD, stable disease; PD, progressive disease; NE, not evaluable; ORR, overall response rate; DCR, disease control rate

#### Toxicities

Table 5 shows all-grade and grade 3–4 toxicities. The most common toxicity was leukopenia. Although most of the toxicity profiles were similar in both arms, grade 3–4 leukopenia and neutropenia were significantly more frequent in the non-SP arm than in the SP arm. All-grade alopecia was less common in the SP arm than in the non-SP arm.

**Table 5:** Toxicities

Adverse effect	SP (N = 159)		Non-SP (N = 157)		p-value
	All grade	Grade 3–4	All grade	Grade 3–4	Grade 3–4
Leukopenia	148	59	153	103	< 0.001
Neutropenia	139	53	146	97	< 0.001
Thrombocytopenia	108	7	86	8	0.797
Anemia	143	23	150	28	0.447
Febrile neutropenia	11	11	16	16	0.321
AST increased	49	0	46	2	0.246
ALT increased	68	2	70	7	0.102
Creatinine increased	42	1	55	0	1.000
Hyponatremia	91	18	81	11	0.242
Nausea	102	3	118	9	0.084
Vomiting	23	2	34	1	1.000
Anorexia	119	16	128	26	0.099
Diarrhea	47	9	30	2	0.060
Esophagitis	107	7	107	6	1.000
Pneumonitis	35	7	36	9	0.618
Alopecia	9	0	51	0	–

SP, S-1 + cisplatin; AST, Aspartate aminotransferase; ALT, Alanine aminotransferase

### Relapse pattern and subsequent treatment

A total of 108 patients in the SP arm and 110 in the non-SP arm had cancer recurrence. As analyzed in Additional file 1: Table S5, the most common relapse site was the primary site. Including central nervous system (CNS) progression, no marked tendency was found in either group. Subsequent chemotherapy was administered to 103 (95.3%) in the SP arm, which was significantly more than in the non-SP arm (90 patients, 81.8%) (p = 0.0098). The subsequent therapies administered following relapse are listed in Additional file 1: Table S6. A total of 28 (28%) in the SP arm and 16 (17.9%) in the non-SP arm received platinum doublet therapy. Docetaxel, with or without other agents, was administered to 23 (23%) in the SP arm and 16 (17.9%) in the non-SP arm. Tyrosine-kinase inhibitors for EGFR, ALK, and ROS-1 were prescribed to 22 (22%) in the SP arm and 17 (19.1%) in the non-SP arm.

OS curves were drawn according to each subsequent treatment. The median OS values of those who received platinum doublet therapy versus those who received a single cytotoxic agent, including DTX, PEM, nab-PTX, S-1 and others, following relapse were 34.6 and 29.2 months, respectively. The corresponding HR between those 2 groups was 0.698 (95% CI 0.429–1.137) (Additional file 2: Fig. S7).

## Discussion

Decisions concerning stage III NSCLC should always be made while incorporating the cumulative perspectives of surgeons, radiologists, and respiratory oncologists due to the heterogeneity of this disease. For locally advanced NSCLC that happens to be unresectable, the only therapy that can possibly provide a cure is concurrent chemoradiotherapy [19]. The purpose of chemotherapy is to destroy occult distant metastases, thus only acting in a supplementary manner, while RT fulfills the main role of eradicating local cancer cells. By choosing the most appropriate regimen that allows for full dosage administration and is as nontoxic as possible, patients may obtain the most benefit from their treatment [20].

Our IPD meta-analysis is the most up-to-date integrated evaluation comparing S-1 to other third-generation anti-cancer medications in combination with cisplatin for stage III NSCLC. Our study found no significant difference between the SP regimen and non-SP regimens in terms of the OS, PFS, or ORR, with SP showing significantly milder hematologic toxicity than non-SP. The median OS of SP was 48.2 months, which was slightly better than the 42.4 months achieved with non-SP regimens, although there was no significant difference. However, the PFS in both groups was similar. The discrepancy in the findings for the OS and PFS can be attributed to the low toxicity of the SP regimen. In our analysis, severe leukopenia and neutropenia were less frequent in SP than in non-SP, which might have led to a reduced need for dose reduction and higher rate of subsequent chemo induction in cases of recurrence. Furthermore, platinum doublet therapy was selected more frequently following relapse for patients who received SP than for those who received non-SP regimens. Since the OS tended to be better for those receiving platinum doublet therapy than for patients who could only tolerate a single cytotoxic agent after relapse, with no significant difference, we suspected that this subsequent treatment difference influenced the overall OS difference between the SP and non-SP arms. In contrast, the higher ratio of delaying treatment and the lower RDI in the SP arm than in the non-SP arm during induction chemoradiotherapy period may have been due to the higher ratio of prolonged grade 1–2 leukocytopenia or neutropenia, which is not severe enough to affect the medication dosage or subsequent treatment. In addition, the ORR and DCR were similar between the groups (69.4% and 92.3% in the SP arm, respectively, and 70.9% and 94.1% in the non-SP arm, respectively).

UFT and S-1 are oral 5-FU derivative medications containing an inhibitor of dehydropyrimidine dehydrogenase (DPD). By blocking DPD, which is a key enzyme breaking down 5FU, these agents are effective even in organs that produce DPD and can overcome cancer resistance [21]. S-1/platinum agent therapy used for gastrointestinal and head/neck malignancies was introduced to NSCLC in 2010 once the randomized phase-3 LETS trial proved its non-inferiority to PTX/CBDCA as a first-line chemotherapy in advanced NSCLC [7]. Lung cancer is known to have a relatively high DPD activity [22], so S1 containing gimeracil, which inhibits DPD, is expected to work better than 5FU alone. In addition to the LETS trial, the randomized phase-3 CATS study published in 2015 comparing S1/CDDP to DTX/CDDP for advanced NSCLC also proved the non-inferiority of S-1 with regard to the OS [8]. The reported toxicities in the S-1 arm were consistently tolerable in both the LETS and CATS trials, including a low rate of grade ≥ 3 leukopenia and febrile neutropenia. The toxicity profile in our study was similar to those in the two previously mentioned trials. Although the S-1 arm tended to show thrombocytopenia more frequently than the non-SP arms in the evaluated series of trials, with the patients allocated to SP in our report showing a rate of 67.9% for all-grade thrombocytopenia while those receiving non-SP showed a rate of 54.7%, more troublesome grade 3–4 thrombocytopenia was rare.

Considering the potent radiosensitizing properties of 5FU and gimeracil, it seems reasonable to expect S1/CDDP and concurrent RT to be beneficial for locally advanced lung cancer [23, 24]. Unrepaired DNA double-strand breaks after radiation therapy result in cell death, and the small molecules that affect DNA damage response can function as radiation sensitizers. 5-FU dysregulates nucleotide pools and causes complex and clustered radiation-induced DNA lesions that can be difficult to repair. The increased long-term survival benefit in the SP arm observed in our study (5-year OS: 44.9% in the SP arm, 26.9% in the non-SP arm) may have been due to this additional radiosensitizing effect exerting a significant effect on the index tumor site as well as possible metastasized lesions.

The 2017 PACIFIC trial was impressive as the only successful trial to prove a statistically significant and clinically meaningful improvement in the PFS and OS by administering the anti-PD-L1 antibody durvalumab as consolidation therapy for one year after CCRT [6]. Consolidation therapy with docetaxel monotherapy, cisplatin-docetaxel, or cisplatin-vinorelbine after CCRT had been explored in earlier randomized phase-3 trials, but all had failed to show any outcome improvement [25–27]. In the PACIFIC trial, the durvalumab arm showed an absolute increase of 10.7% in the 2-year survival rate compared to placebo, which changed our perspective concerning how CCRT regimens should be employed. In our study, 17.9% of S-1/CDDP patients required dose reduction during CCRT, in contrast to 27.5% of patients receiving non-SP regimens (p = 0.034), and 95.3% of those in the SP arm were able to progress to second-line chemotherapy in cases of relapse, compared to 81.8% in the non-SP arm (p = 0.001). We assume that the significantly lower dose reduction rate and higher chemotherapy shifting rate in the SP arm than in the non-SP arm indicate that patients tolerated this regimen well and maintained a good status. Theoretically, the earlier an immune-checkpoint inhibitor is initiated after RT, the better a response we can expect, as RT can help release tumor-related antigens and enhance the antigen presentation, helping immune system cells block the checkpoint [28, 29]. The better the status in which patients end CCRT, the better their chances of surviving and the more quickly they can shift to upcoming ICI consolidation therapy.

According to a multivariate analysis, the only covariate that demonstrated an OS influence was age; none of the other factors, including the treatment (OS/Others), were influential. The HR of patients ≥ 70 years old versus those < 70 years old was 1.638 (95% CI: 1.096–2.449, p = 0.016). Elderly patients are at a higher risk of developing severe treatment-related toxicities than younger patients because they tend to have more comorbidities, which can interrupt or prevent treatment completion. However, previously reported randomized controlled trials and a meta-analysis determined that the addition of chemotherapy to RT alone undeniably benefits patients ≥ 70 years old [30, 31]. Therefore, we believe that there is some benefit to developing a treatment specifically designed for elderly patients.

With regard to the usefulness of S-1 for squamous cell lung cancer, our meta-analysis also indicated that cisplatin and S-1 combined with thoracic RT was a good treatment option for non-squamous unresectable stage III NSCLC. Pemetrexed, in addition to S-1, is a key cytotoxic agent that disrupts folate-dependent cell replication by inhibiting thymidylate synthetase enzymes involved in purine and pyrimidine synthesis. Although pemetrexed inhibits broader folic acid-associated enzymes than S-1, its toxicities are usually tolerable with the supplementation of folic acid and vitamin B12 one week prior to the drug administration. A subset analysis of previously reported phase-3 trials—one comparing CDDP/pemetrexed to CDDP/gemcitabine in the first-line setting and another comparing pemetrexed to docetaxel in a second-line treatment course—showed that pemetrexed significantly prolonged the OS in cases of non-squamous histology versus squamous [32, 33]. Since stage III NSCLC has a high recurrence rate, sparing pemetrexed for second-line treatment may be beneficial, as it might result in a longer survival time in cases of non-squamous cell lung cancer.

Several limitations associated with the present study warrant mention. First, the three randomized control trials that were combined and analyzed together were conducted along different timelines. Due to the rapid development of chemotherapy for subsequent-line treatment as well as staging techniques, including fluorodeoxyglucose-positron emission tomography-computed tomography, there is likely a gap in the OS results among these three randomized control trials. However, we strongly believe that this integrated meta-analysis of three trials is reasonable and meaningful because the HR for the OS in each trial showed no heterogeneity. Second, testing for driver mutations was not mandatory at enrollment in any of the studies and was only examined in cases of relapse. Because lung cancer with driver mutations has a better prognosis than that without such mutations, the OS may have been biased depending on the status. However, TKIs for EGFR, ALK, and ROS-1 were administered to almost the same percentage of patients in each arm following relapse, as previously mentioned. We may thus reasonably suspect that both arms had similar ratios of patients with driver mutations. Third, durvalumab was not given in our study, as all randomized control trials were conducted before the PACIFIC study. Although we were able to examine the use of ICI as second-line therapy after relapse, with the rates being about the same in both arms, whether or not ICIs were included in further subsequent treatment was not examined. Finally, the three randomized control trials were all performed in Japan. Because S-1 is used mostly in Asian countries at present, we were unable to collect randomized control trial data from all over the world at this time. Due to the bias in ethnicity, our study findings should be carefully interpreted. The safety profiles may differ depending on the geographic region, given the findings of previous studies conducted in North America and Europe [34–36].

## Conclusion

There was no significant difference found in the OS, PFS, or ORR between S-1/cisplatin and VNR, PEM, or DTX/cisplatin as a CCRT regimen for locally advanced NSCLC. SP is a well-tolerated regimen due to its acceptable toxicity and treatment compliance.

## Supplementary Information

**Additional file 1.** Supplementary tables.

**Additional file 2.** Supplementary figure.

## Data Availability

The data that support the findings of this study are available from the corresponding author (Y.T.) upon reasonable request.
