# Supplementary material for: Concurrent chemoradiotherapy with cisplatin + S-1 versus cisplatin + other third-generation agents for locally advanced non-small-cell lung cancer: a meta-analysis of individual participant data
Source: BMC Pulm Med. 2022 Jan 9;22:31. doi: 10.1186/s12890-022-01828-z (PMC8744285; doi:10.1186/s12890-022-01828-z)
Supplement: Supplementary file 1 — Additional file 1. Supplementary tables. [file 12890_2022_1828_MOESM1_ESM.docx]

Supporting information

**Table S1. Relative dose intensity of each drug**

|  |  | courses | SP | non-SP | p |
| --- | --- | --- | --- | --- | --- |
| RDI (average, 95%CI) | CDDP | 1 to 2 | 91.3 (89.4-93.2) | 95.1 (93.2-97.0) | 0.007 |
|  | S-1 or the others | 1 to 2 | 90.5 (88.3-92.7) | 93.7 (92.2-95.3) | 0.019 |
|  | CDDP | 3 to 4 | 69.5 (63.7-75.3) | 74.9 (69.6-80.2) | 0.173 |
|  | S-1 or the others | 3 to 4 | 67.8 (62.1-73.5) | 73.8 (68.6-79.0) | 0.129 |

RDI, relative dose intensity; CDDP, cisplatin; SP, S-1+cisplatin.

**Table S2. Reasons for delaying the treatment**

|  | SP(n=114) | non-SP(n=97) | Fisher's test  p |
| --- | --- | --- | --- |
| Leukopenia or Neutropenia | 72 | 48 | 0.052 |
| Thrombocytopenia | 8 | 4 | 0.553 |
| Fever | 1 | 5 | 0.096 |
| Creatinine increased | 3 | 7 | 0.192 |
| Non-hematologic AE greater than Gr3 | 3 | 5 | 0.475 |
| Not related to AE | 47 | 44 | 0.579 |

AE, adverse events.

**Table S3. The OS and PFS**

|  | SP (N=159) | Non-SP (N=157) |
| --- | --- | --- |
| Median OS (months) | 48.2 | 42.4 |
| (95% CI) | 36.2-NR | 34.6-65.6 |
| Two-year OS (%) | 74.7 | 67.5 |
| (95% CI) | 67.4-80.9 | 59.7-74.4 |
| Five-year OS (%) | 44.9 | 26.9 |
| (95% CI) | 32.8-57.6 | 10.4-53.9 |
| Number of events | 68 | 75 |
| Median PFS (months) | 12.8 | 14.1 |
| (95% CI) | 11.2-15.9 | 12.1-16.4 |
| Two-year PFS (%) | 33.9 | 35.2 |
| (95% CI) | 26.8-41.8 | 27.9-43.3 |
| Number of events | 108 | 110 |

SP, S-1+cisplatin; OS, overall survival; PFS, progression-free survival; NR, not reached.

**Table S4. Multivariate analysis results**

|  | HR | 95%CI | p-value |
| --- | --- | --- | --- |
| WJOG; SP vs Others | 0.775 | 0.446-1.348 | 0.367 |
| TORG; SP vs Others | 0.918 | 0.515-1.639 | 0.336 |
| SPECTRA; SP vs Others | 0.959 | 0.526-1.746 | 0.803 |
| SP vs non-SP | 0.881 | 0.631-1.228 | 0.454 |
| age ≧70/<70 | 1.638 | 1.096-2.449 | 0.016 |
| Gender; female vs male | 0.955 | 0.581-1.569 | 0.855 |
| Smoking; ever vs never | 1.135 | 0.636-2.027 | 0.669 |
| PS; 1 vs 0 | 1.076 | 0.760-1.523 | 0.679 |
| Histology; non-adeno vs adeno | 1.344 | 0.920-1.964 | 0.126 |
| stage; ⅢB vs ⅢA | 1.261 | 0.903-1.759 | 0.173 |

SP, S-1+cisplatin; HR, hazard ratio; PS, performance status.

**Table S5. Relapse site**

|  | SP (n=159) | Non-SP (n=157) | Fisher's test p-value |
| --- | --- | --- | --- |
| relapse | 108 | 110 |  |
| primary site | 41 | 46 | 0.529 |
| lung metastasis | 25 | 21 | 0.633 |
| pleural effusion | 8 | 14 | 0.191 |
| regional lymph nodes | 27 | 22 | 0.535 |
| CNS | 23 | 22 | 1.000 |
| bone | 16 | 10 | 0.306 |
| liver | 5 | 7 | 0.571 |
| adrenal gland | 8 | 12 | 0.365 |
| others | 9 | 7 | 0.798 |

SP, S-1+cisplatin; CNS, central nervous system

**Table S6. Second-line therapy following relapse**

|  |  | SP (n=100) | Non-SP (n=89) | p-value |
| --- | --- | --- | --- | --- |
| subsequent chemotherapy | platinum doublet | 28 | 16 | 0.122 |
|  | DTX with or without other agents | 23 | 16 | 0.472 |
|  | other single cytotoxic agent (PEM,S-1,GEM,VNR,AMR,nab-PTX) | 11 | 24 | 0.008 |
|  | TKI (EGFR, ALK, ROS-1) | 22 | 17 | 0.720 |
|  | immune-checkpoint inhibitors | 5 | 9 | 0.266 |
|  | clinical trial participation | 2 | 1 | 1.000 |
| surgery |  | 9 | 12 | 0.361 |
| pleural drainage |  | 1 | 0 | 1.000 |
| bronchoscopic coagulation |  | 0 | 2 | 0.220 |
| radiation |  | 43 | 42 | 0.661 |

SP, S-1+cisplatin; DTX, docetaxel; PEM, pemetrexed; GEM, gemcitabine; VNR, vinorelbine; AMR, amrubicin; PTX, paclitaxel; TKI, tyrosine kinase inhibitor; EGFR, epidermal growth factor receptor; ALK, anaplastic lymphoma kinase; ROS-1, c-ros proto-oncogene 1.

**Fig S7. Overall survival (OS) curves by subsequent chemotherapy.**

DTX, docetaxel; PEM, pemetrexed; PTX, paclitaxel; TKI, tyrosine kinase inhibitor; ICI, immune-checkpoint inhibitor.
