# Supplementary figures and images for: Concurrent chemoradiotherapy with cisplatin + S-1 versus cisplatin + other third-generation agents for locally advanced non-small-cell lung cancer: a meta-analysis of individual participant data
Source: BMC Pulm Med. 2022 Jan 9;22:31. doi: 10.1186/s12890-022-01828-z (PMC8744285; doi:10.1186/s12890-022-01828-z)

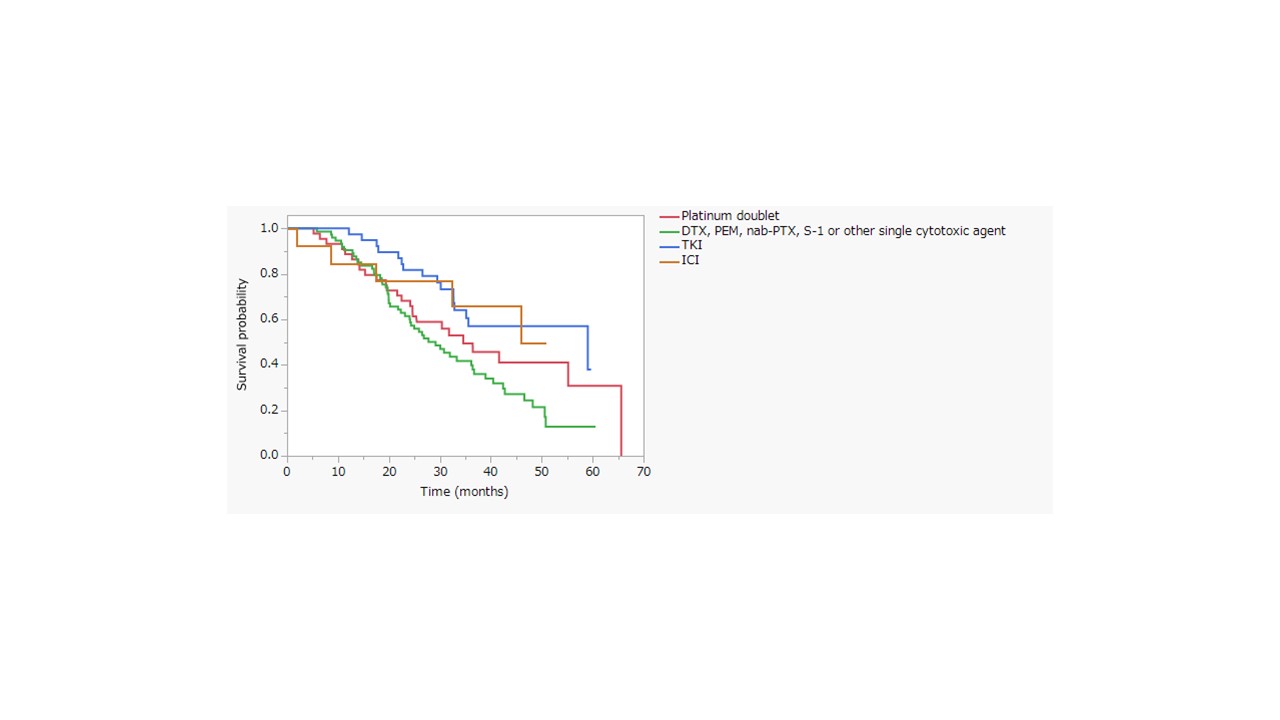

Supplement: Supplementary file 2 — Additional file 2. Supplementary figure. [file 12890_2022_1828_MOESM2_ESM.jpg]
